# The potential of antioxidant-rich Maoberry (*Antidesma bunius*) extract on fat metabolism in liver tissues of rats fed a high-fat diet

**DOI:** 10.1186/s12906-019-2716-0

**Published:** 2019-11-04

**Authors:** Chattraya Ngamlerst, Arunwan Udomkasemsab, Ratchanee Kongkachuichai, Karunee Kwanbunjan, Chaowanee Chupeerach, Pattaneeya Prangthip

**Affiliations:** 1 Institute of Nutrition, Mahidol University, Nakhon Pathom, 73170 Thailand; 2 Department of Tropical Nutrition and Food Science, Faculty of Tropical Medicine, Mahidol University, Bangkok, 10400 Thailand

**Keywords:** Liver tissue, *Antidesma bunius*, Maoberry, High fat diet

## Abstract

**Backgound:**

Obesity and dyslipidemia are major risk factors associated with non-alcoholic fatty liver disease (NAFLD). NAFLD refers to the accumulation of fat in more than 5% of the liver without alcohol consumption. NAFLD is the most common liver disease and is rapidly becoming a global public health problem. Maoberry (*Antidesma bunius*) is a fruit rich in antioxidants, especially phenolic compounds, which are reported to have benefits for patients with NAFLD.

**Methods:**

We evaluated the effect of Maoberry extract on fat metabolism in liver tissues of high fat diet–induced rats. Five (5) groups (*n* = 12) of male Sprague-Dawley (SD) rats were divided into those given a high fat diet with no treatment (HF), different dosages of Maoberry extracts (0.38 [ML], 0.76 [MM) and 1.52 [MH] g/kg body weight) and 10 mg/kg statin (STAT). The rats were fed a high fat diet for 4 weeks to induce obesity and subsequently continued more for 12 weeks with treatments of Maoberry extracts or STAT. The levels of triglyceride, liver enzymes, oxidative stress and inflammation markers, triglyceride synthesis regulators, and pathology of the liver in high fat diet-induced rats were investigated.

**Results:**

Feeding Maoberry extract in MH groups resulted in decreasing levels of serum alanine aminotransferase (ALT), liver triglyceride, liver thiobarbituric acid reactive substances (TBARS) and mRNA expression of tumour necrosis factor (TNF)-α, interleukin (IL)-6, glycerol-3-phosphate acyltransferase (GPAT)-1 and acetyl-coenzyme A carboxylase (ACC) compared with the HF group (*P* < 0.05). Moreover, histopathological study of the liver showed reduced fat droplets in the Maoberry extract treatment groups, especially in MH groups and STAT treatment groups.

**Conclusions:**

The improvements of fat metabolism in liver tissues of rats fed a high-fat diet were observed in Maoberry extracts treatment groups. The underline mechanism that link to fat metabolism might be through the process accompanied with down-regulated the gene expression of key enzymes of lipid production, antioxidant activity, and anti-inflammation properties of Maoberry extracts which contains high levels of phenolic and flavonoid compounds.

## Background

The global prevalence of obesity in adults has dramatically increased over a period of time by almost three times from 3.2% in 1975 to 13% in 2016 [1]. Thailand is one of the highest prevalence of obesity in Asia. In 2009, the prevalence of obesity was approximately 35%, increasing more than 2.5 times compared to 1991 [2], which 66.5% of the same population group has abnormal blood cholesterol [3]. Obesity and dyslipidemia are major risk factors associated with non-alcoholic fatty liver disease (NAFLD). Fat accumulation in the liver by more than 5% without significant alcohol consumption represents NAFLD [4]. It is the most common liver disease worldwide, although there has not been a report of actual fatty liver levels due to different clinical and histological forms [5]. NAFLD causes increasing oxidative stress, tissue inflammation, and hepatocytes malfunction. Simple steatosis can progress to nonalcoholic steatohepatitis (NASH), fibrosis, cirrhosis, and ultimately hepatocellular carcinoma [6]. Consumption of a high fat diet causes increasing body weight and enlargement of internal organs, including the liver, as well as dyslipidemia and obesity in rats [7–9]. Feeding high fat diet to rats were able to attribute characteristics hypercholesterolemia which is relevance to human biology [8–12]. A high fat diet was therefore an option in this study.

Maoberry (*Antidesma bunius*) have been reported to have the antioxidant activity and capacity, due to rich in polyphenol, especially anthocyanin [13, 14]. There are many studies on anthocyanin for health-promoting attributes. The health benefits include improved regulation of blood sugar levels, action against hypercholesterolemia and reduced risk for cardiovascular disease due to the antioxidant properties [15, 16]. Previous reports suggest that fruits rich in flavonoids and phenolic acids have been shown to improve the features of NAFLD, such as oxidative stress, dyslipidemia, liver steatosis, and inflammation in rodents [10–12]. Our previous study reported the health benefits of Maoberry around four to sixteen portions a day could against in hypercholesterolemia and progression of cardiac tissue deterioration in rats [8, 9]. Supplement rats with Maoberry might be somehow useful on fat metabolism in liver tissues. We investigated the effect of Maoberry extract consumption on fat metabolism in liver tissues of rats fed a high fat diet.

## Methods

### Maoberry extract preparation

Maoberry fruit (*Antidesmabunius spp*.) was purchased from local farm in Sakon Nakhon province, North-eastern Thailand (Geocode as 4715) during August–September in year 2016. Maoberry fruits in this study were identified and confirmed by Assistant Dr. Prof. Pornprapha Chunthanom. A voucher specimen of this material has been deposited in Faculty of Natural Resources, Rajamangala University of Technology Isan, Sakon Nakhon, Thailand. Maoberry fruits with black color without visible injuries were used for preparing of Maoberry extract. Briefly, Maoberry fruits were washed and homogenized using a blender (TEFA blenforce, TEFAL, Bangkok, Thailand). The 40 mesh filter was employed to eliminate seeds and marc. Total soluble solid contents of juice were measured approximately 18% Brix using a hand-held refractometer (Master, Atago Co, Ltd., Tokyo, Japan) and concentrated to 60% concentration (v/v) using rotary vacuum evaporator (*BUCHI rotavapor R-200, BUCHI*, Flawil, Switzerland). Extracts were packed and preserved in several airtight bottles at − 20 °C until used.

### Anthocyanidins contents determination in Maoberry extract

Anthocyadins (cyanidin and peonidin) contents were analysed as described by Kongkachuichai et al. [17]. One gram Maoberry juice was extracted with 10 mL aqueous solution consisting of water, methanol and concentrated hydrochloric acid (HCl; 33:50:17: v/v/v) and sonicated for 20 min (Branson, 2510, Danbury, CT, USA). Then, the extracts were heated in a boiling water bath (Memmert, Duesseldorf, Germany) for 1 h before cooling immediately in an ice bath. The solution *layer* was separated by *centrifuging* at 4 °C, 2000 rpm for 30 min (HIMAC centrifuge, CR5BB2, HITACHI, Tokyo, Japan) and filtrated through a 0.45 μm membrane filter (Chrom Tech®, Milford, MA, USA) before injection to high performance liquid chromatography (HPLC) system. a HPLC analysis system were performed by using a C18 column (Waters NovaPac C18, 100 × 4.6 mm; Waters Corporation, Milford, MA, USA) equipped with a Waters 515 pump (Water Corporation) and Jasco UV 975 detector (Jasco International, Co., Ltd., Tokyo, Japan). The mobile phase was consisted of 0.4% trifluoroacetic acid (TFA) in water and 0.4% TFA in acetonitrile at a ratio of 18:82 with a flow rate of 0.9 mL/min. Eluate was monitored at 530 nm. The results were expressed as milligrams cyanidin equivalents per 100 g Maoberry extract (mg CE/100 g).

### Total polyphenol contents determination in Maoberry extract

Total polyphenol contents were estimated following the Folin-Ciocalteu method of Baba and Malik [18] with some modifications. Ten microliter sample was mixed with 150 μL of distilled water in 96-well microplate and mixed thoroughly with 25 μL Folin-Ciocalteu reagent (Sigma-Aldrich Corp., St. Louis, MO, USA) for 3 min. Then, the mixture was added to 100 μL 20% (w/v) sodium carbonate (Ajax Finechem, Auckland, New Zealand) and allowed to incubate at room temperature for 60 min in the dark. The absorbance of blue-complex was measured at 650 nm using a microplate reader (Tecan Sunrise, Männedorf, Switzerland). Gallic acid monohydrate (125, 250, 500, 750, 1000 μL/mL; Sigma-Aldrich Corp.) and deionised water were used as standard and blank, respectively. The total polyphenol contents of samples were expressed as milligram of gallic acid equivalents (GAE) in 100 g sample.

### Total flavonoid contents determination in Maoberry extract

Total flavonoid contents were determined by the aluminium chloride colourimetric method of Baba and Malik [18] with some modifications. Sample (1.5 μL) was mixed with 28.5 μL methanol (Sigma-Aldrich Corp.), 120 μL distilled water, 9 μL 5% NaNO_2_ (Sigma-Aldrich Corp.) and 9 μL of 10% AlCl_3_ (GAMMACO, Nonthaburi, Thailand) in 96-well microplates. The mixture was allow at room temperature for 5 min in the dark. Then, 60 μL of 1 M sodium hydroxide (NaOH; Merck Millipore, Darmstadt, Germany) and 72 μL distilled water were added. The mixture was allowed to incubate at room temperature more for 15 min, and was measured at the absorbance of 410 nm using a microplate reader. Quercetin (100, 200, 400, 600, 800, 1000 μL/mL; Sigma-Aldrich Corp.) was used as standard. Total flavonoid contents of samples were expressed as milligram of quercetin equivalents (QE) in 100 g sample.

### Animals

This study was approved by the Ethics Committee on Animal Experimentation of the Faculty of Tropical Medicine – Animal Care and Use Committee (FTM-ACUC), Mahidol University, Bangkok, according to the Animal Experimentation (FTM-ACUC 002/2017). Five week-old Male Sprague-Dawley rats weighting 130–160 g were purchased from the National Laboratory Animal Center at Salaya Campus, Mahidol University. All rats were housed at the Faculty of Tropical Medicine, Mahidol University in accordance with the rules and regulations of FTM-ACUC of the Laboratory Animal Science Center. All rats were closely monitored the welfare-rated assessments throughout the experiment by researchers and veterinarians of laboratory animals. After allowing the rats to become accustomed to a new environment for a week with a normal rodent diet given in a metal container and a bottle of drinking water, the rats were housed in plastic cages (two rats/cage) with an open top in an atmosphere of 55 ± 5% relative humidity, 22 ± 2 °C and a 12:12 light-dark cycle–controlled room. After 1 week of adaptation phase, all rats received a high fat diet (5.34 kcal/g diet consisting of carbohydrate 22%, fat 59% and protein 19% base on nutrition distribution) for 4 weeks to induce obesity and subsequently continued more for 12 weeks with treatments of Maoberry extracts or statin. During treatment, rats were divided randomly into five groups with 12 rats in each group. The number of sample size of 12 was calculated from related researches that employ high fat models [19–21]. The groups were classified as following: high fat diet (HF), high fat diet with Maoberry extract 0.38 g/kg BW (ML), high fat diet with Maoberry extract 0.76 g/kg BW (MM), high fat diet with Maoberry extract 1.52 g/kg BW (MH) and high fat diet with 10 mg/kg of statin (STAT). Maoberry extract and STAT were gavaged every alternate day around 10.00 am according to body weight of each rat for 12 weeks. At the end of the experiment, all rats were fasted for 16 h. The rats were euthanized with carbon dioxide inhalation according to the protocol of the Laboratory Animal Science Unit, Faculty of Tropical Medicine, Mahidol University. In short*,* each rat was normally placed in 25 l polycarbonate chamber. Then, emitting CO_2_ into chamber at a flow rate of about 5.5–7.5 L/min until the rat was unconscious. The flow of CO_2_ continued for at least 60 s to ensure that the breath was not seen before removing the rat from the chamber. Whole blood was drawn from the inferior vena cava and immediately sent for further analysis of lipid profile and liver enzymes functions (central laboratory of National Healthcare Systems Co., Ltd., Bangkok, Thailand). After blood collection and gross observation, the liver was collected, trimmed, washed with normal saline, wiped with filter paper and weighed. Tissue was frozen immediately in liquid nitrogen and stored at − 80 °C until further analysis. Separated liver was immersed in 10% neutral buffered formaldehyde overnight, then drained of the previous formaldehyde and the solution filled again repeatedly for histological observation.

### Histological analysis

To observe the size and number of fat globules in the liver, the liver tissue samples were fixed in 10% buffered formalin and embedded in paraffin. Tissue sections (5 μm) were cut with a microtome and mounted on microscope slides. The slides then were stained with haematoxylin and eosin and photographed via × 20 and × 100 objective lenses (Olympus BX43 Microscope and Olympus DP20 Microscope Camera, Olympus America, Inc., New York, USA).

### Liver triglyceride (TG) contents

Liver TG levels were determined using the Triglyceride Colorimetric Assay Kit (Cayman Chemical Company, MI, USA) according to the manufacturer’s instructions. Briefly, 400 mg liver tissues were homogenised with 2 mL diluted standard diluent. The mixture was centrifuged at 10,000×g at 4 °C for 10 min. Then, 10 μL supernatant was transferred to the well of the microplate. Afterward, 150 μL diluted enzyme buffer solution was mixed with that supernatant in the well. The mixture was allowed to incubate at room temperature for 15 min. The absorbance was measured at 550 nm.

### Thiobarbituric acid reactive substances (TBARS) assay in serum and liver tissue

Malondialdehyde (MDA) of samples was determined with the OxiSelectTM TBARS Assay Kit (Cell Biolabs, Inc., San Diego, CA, USA). A 100 μL sample was mixed with 100 μL 10% sodium dodecyl sulphate (SDS) lysis solution in a microcentrifuge tube and incubated for 5 min at room temperature. Then, 1X 2-thiobarbituric acid (TBA) diluent (250 μL) was added. The mixture was allowed to incubate at 100 °C for 50 min. After that, the tube was removed and placed into an ice bath for 5 min. The tube was centrifuged at 9000 rpm, 4 °C for 15 min. The supernatant of sample (200 μL) was transferred to a 96-well microplate. Absorbance was measured at 532 nm. MDA standard solution was used as standard. TBARS of liver supernatant were expressed as nanomole of MDA equivalents per gram protein.

### Gene expression analysis by quantitative reverse transcriptase-polymerase chain reaction (qRT-PCR)

Total RNA was isolated from liver tissue using TRIzol® reagent (Life Technologies, Carlsbad, CA, USA). The yield of RNA was determined using the NanoDrop 2000 Spectrophotometer (Thermo Fisher Scientific, Waltham, MA, USA). First-strand cDNA was prepared from total RNA by mixing 1 μL 10 mM dNTP Mix (Thermo Fisher Scientific) with 2 μg total RNA sample. Then, 1 μL 0.5 μg/μL Oligo (dT) primer (Thermo Fisher Scientific) was added. Diethyl pyrocarbonate (DEPC) water was added into the mixture to make the volume up to 15 μL. The sample was incubated at 65 °C for 5 min (ProFlex™ 3 × 32-Well PCR System; Thermo Fisher Scientific) and placed on ice for 1 min. Afterwards, 1 μL 200 U/μL Reverse Aid Reverse Transcriptase (Thermo Fisher Scientific) and 4 μL 5Χ reaction buffer were added. The sample was incubated at 42 °C for 60 min and incubated continued at 70 °C for 5 min. Finally, the sample was stored at − 20 °C for further studies. The sequences of primers (Bio Basic, Inc., New York, NY, USA), size and qRT-PCR conditions are shown in Table 1. qRT-PCR analysis was performed with a LightCycler® 480 SYBR Green I Master (Roche Molecular Systems, Inc., Mannheim, Germany) according to the manufacturer’s protocol. The highly specific measurement of cDNA was performed for tumour necrosis factor (TNF)-α, interleukin (IL)-6, glycerol-3-phosphate acyltransferase (GPAT)-1, acetyl-coenzyme A carboxylase (ACC), sterol regulatory element binding protein (SREBP)-1c and β-actin using the LightCycler® 480 Instrument II (Roche Molecular Systems). Each sample was run and analysed in duplicate. The crossing point (Cp) and melting temperature (Tm) were analysed using the LightCycler® 480 software release 1.5.0 version 1.5.0.39 (Roche Molecular Systems). The relative quantification value was determined by Livak’s method [22] according to the following equations:

$$ \Delta Cp(test)= Cp\left( target, test\right)- Cp\left( ref, test\right)\ (1). $$

$$ \Delta Cp(calibrator)= Cp\left( target, calibrator\right)- Cp\left( ref, calibrator\right)\ (2). $$

$$ (1)--(2);\Delta \Delta Cp=\Delta Cp(test)-\Delta Cp(calibrator) $$

$$ {2}^{-\Delta \Delta Cp}= Normalised\ expression\ ratio. $$

**Table 1:** Primer sequences and conditions for qRT-PCR

mRNA	Primer sequence	Product size (bp)	Ta (°C)
β-actin	F: TCATGAAGTGTGACGTTGACATCC R: GACTGTTACTGAGCTGCGTTTTAC * NM_031144.3	311	53
TNF-α	F: ACTGAACTTCGGGGTGATTG R: GTCGTAGCAAACCACCAAGC * HQ201305.1	153	56
IL-6	F: ATATGTTCTCAGGGAGATCTTGGAA R: GTGCATCATCGCTGTTCATACA *NM_031512	80	60
GPAT-1	F: AGACACAGGCAGGGAATCCAC R: AATTCCCGGAGAAGCCCAG *AF021348	103	56
ACC	F: GCCCACTTTCTTCTATCACGCTAA R: GAAGACGGCAGCATGAACTG *XM_017598248.1	159	56
SREBP-1c	F: CAGAGGGACTACAGGCTGAGAAAG R: CACGTAGATCTCTGCCAGTGTTG *NM_001276708.1	204	56

* NCBI Reference Sequence, bp indicated base pair

### Statistics analysis

After testing the normality with the Kolmogorov–Smirnov test, data of experimental groups were analysed using one-way analysis of variance (ANOVA), compared with Tukey’s test. Data are showed as the mean ± the standard error of the mean (SEM). Significant differences were considered at *P* < 0.05, and higher significance at *P* < 0.01 and *P* < 0.001, respectively. All measurements were performed using Statistical Package for the Social Science for Windows (SPSS version 13.0, IBM Corporation, Armonk, NY, USA).

## Results

### Polyphenolic composition and total antioxidant capacity of Maoberry extract

Maoberry extract contained total phenolics, total flavonoid contents and anthocyanidins at 317.15 mg GAE/100 g, 318.25 mg QE/100 g, and 0.05 mg CE/100 g, respectively (Table 2).

**Table 2:** Chemical composition of Maoberry extract

Chemical composition	Contents/ levels
Total polyphenols (mg GAE/100 g)	317.15 ± 1.39
Total flavonoid contents (mg QE/100 g)	381.25 ± 15.79
Anthocyanins (mg/100 ml)	
-Cyanidin	1.94 ± 0.03
-Peodinin	0.36 ± 0.02

Values are expressed as means ± SEM

### Effect of Maoberry extract or STAT on the levels of liver and serum lipid peroxidation in NAFLD rats fed high fat diets

**Fig. 1:**
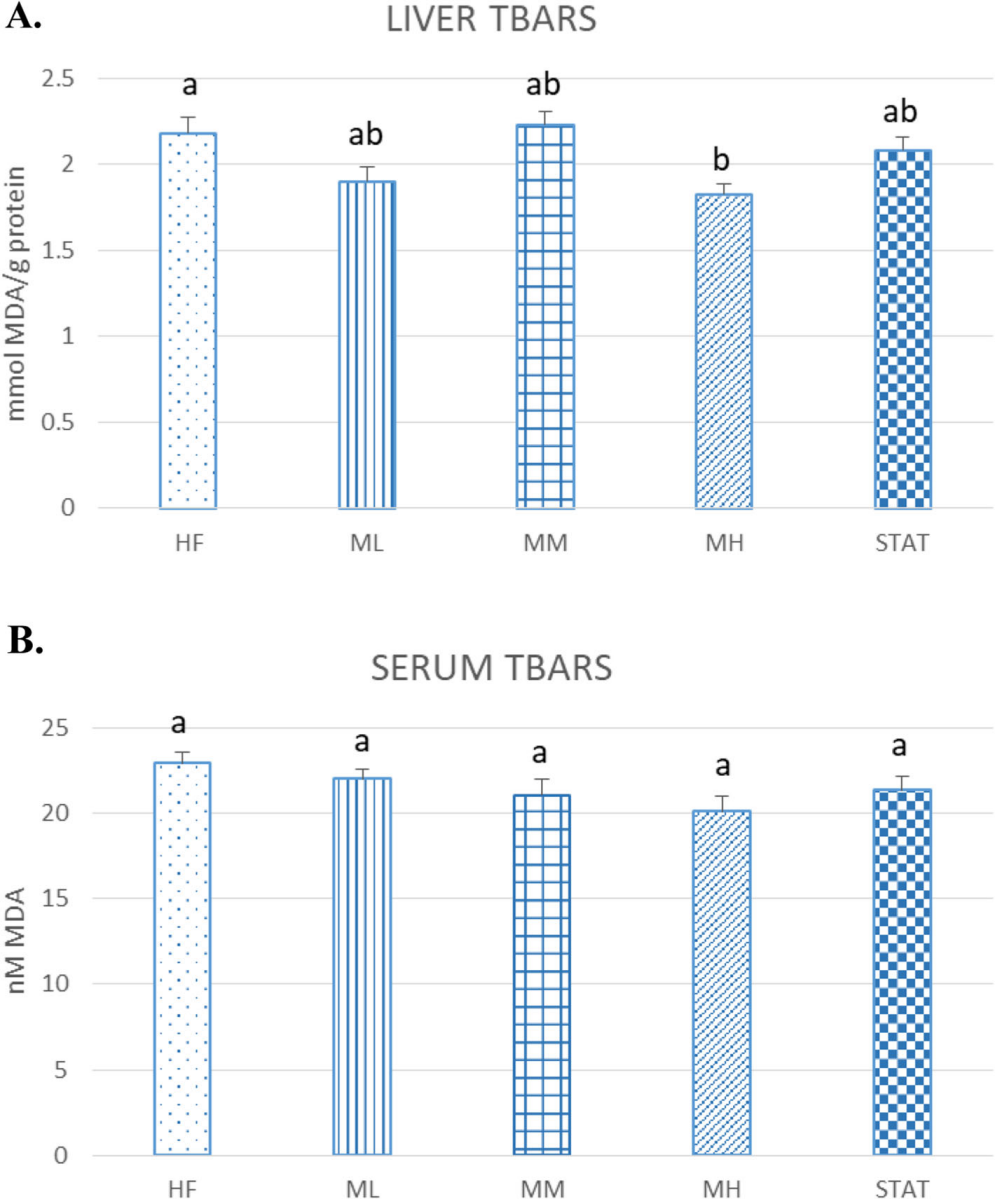
Effect of Maoberry extract on (**a**) liver and (**b**) serum lipid peroxidation levels (*n* = 12). The groups were classified as follows: high fat diet with no treatment (HF); three dosages of Mao-Luang extract (0.38 [ML], 0.76 [MM] or 1.52 [MH] g/kg BW) and 10 mg/kg of STAT. Values are expressed as means ± SEM. Different letters indicate significant difference among group at *P* < 0.05

To evaluate the effect of Maoberry extract supplemention in liver tissue and serum, TBARS values, indicating oxidative stress by increased lipid peroxidation, were measured. Maoberry extract resulted in decreased liver TBARS values in the ML and MH but not the MM groups. The decrease in the MH group (16.51%) was significant compared with the HF group (*P* = 0.018). In contrast, there was no significant difference in serum TBARS levels among the rat groups (Fig. 1).

### Effect of Maoberry extract or STAT on hepatic oxidative stress markers, inflammation markers and TG synthesis regulators

**Fig. 2:**
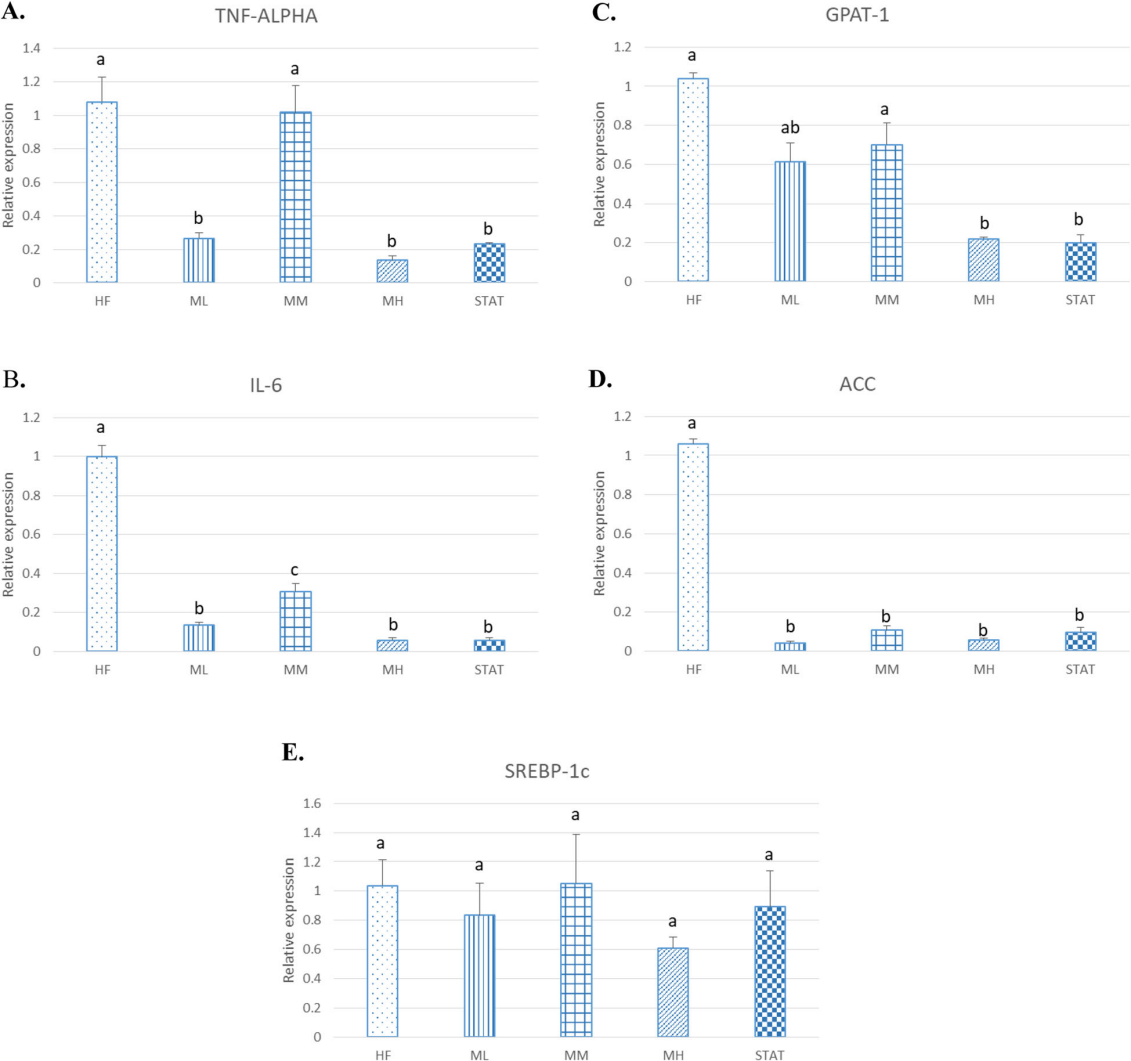
Hepatic (**a**) TNF-α, (**b**) IL-6, (**c**) GPAT-1, (**d**) ACC and (**e**) SREBP-1c expression normalised to β-actin (*n* = 12). The groups were classified as follows: high fat diet with no treatment (HF); three dosages of Mao-Luang extract (0.38 [ML], 0.76 [MM] or 1.52 [MH] g/kg BW) and 10 mg/kg of STAT. Values are expressed as means ± SEM. Different letters indicate significant difference among groups at *P* < 0.05

Another liver tissue sample was subjected to two-step qRT-PCR for examination of changes in mRNA expression of oxidative stress and inflammation markers and TG synthesis regulators, such as TNF-a, IL-6, GPAT-1, ACC and SREBP-1c, which were associated closely with NAFLD (Fig. 2).

Compared with the HF group, administration of Maoberry extract at three different dosages and of STAT in rats fed a high fat diet showed dramatic downregulation of hepatic TNF-α mRNA expression by 75.5, 5.8, 87.4 and 87.7% for the ML, MM, MH and STAT groups, respectively, as well as significantly decreased hepatic expression of IL-6 by 86.5, 69.3, 94.2 and 94.2%, respectively, (*P* < 0.05).

Maoberry supplementation and STAT treatment reduced hepatic levels of GPAT-1, ACC and SREBP-1c compared with the HF controls. Hepatic mRNA expression of GPAT-1, which is a rate-limiting enzyme of triacylglycerol biosynthesis, was decreased significantly in the MH and STAT groups by 78.9 and 80.8%, respectively (*P* < 0.05). Hepatic gene expression of ACC, which is a rate-controlling enzyme of lipogenesis, was decreased significantly after treatment by 96.0, 89.8, 94.6 and 90.9% for the ML, MM, MH and STAT groups, respectively (*P* < 0.05). Although hepatic gene expression of SREBP-1c appeared to be diminished in Maoberry extract- and STAT-treated rats, the effects were not statistically significant.

### Effect of Maoberry extract or STAT on the levels of liver TG, and liver enzymes

**Fig. 3:**
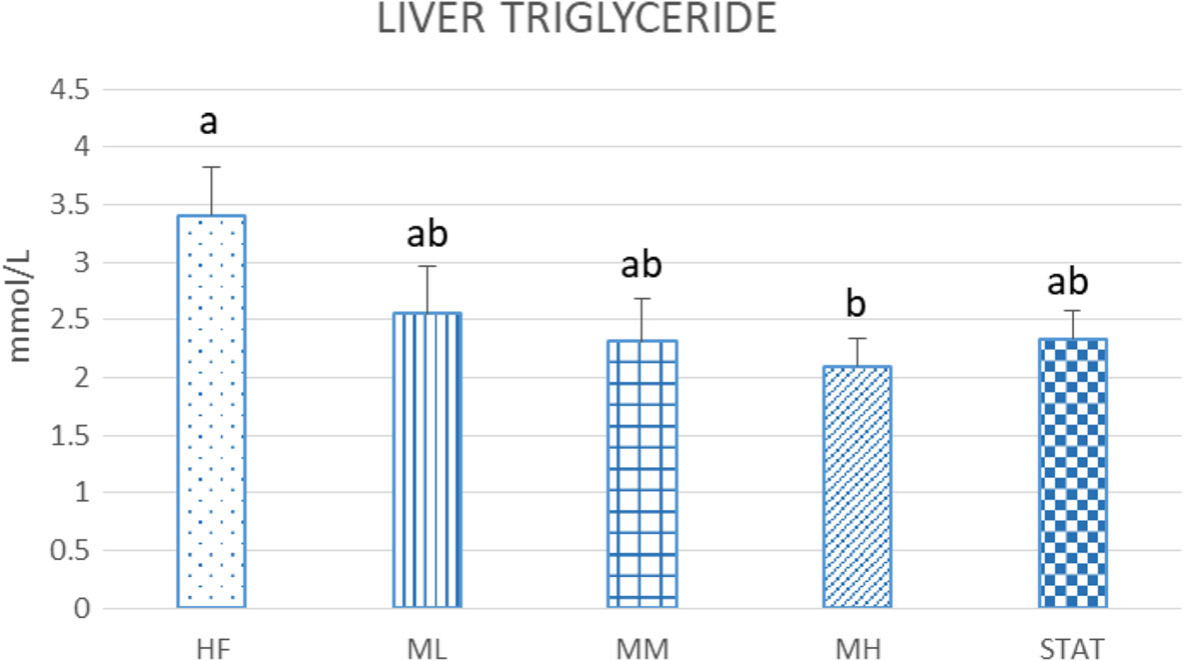
Effect of Maoberry juice on the levels of liver TG (*n* = 12). The groups were classified as follows: high fat diet with no treatment (HF); three dosages of Mao-Luang extract (0.38 [ML], 0.76 [MM] or 1.52 [MH] g/kg BW) and 10 mg/kg of STAT. Values are expressed as means ± SEM. Different letters indicate significant difference among groups at *P* < 0.05

Compared with the HF group (3.45 ± 0.33 mmol/L), liver TG levels of the MH group (2.09 ± 0.20 mmol/L) were decreased significantly by 38.7% (*P* = 0.048), but no statistically significant difference was found among the ML, MM and STAT groups (2.55 ± 0.38, 2.31 ± 0.35 and 2.34 ± 0.19 mmol/L, respectively (Fig. 3).

There was no significant difference in serum AST level within the experimental groups. However, compared with the HF group, the ML, MM and MH groups had lower serum AST levels after treatment for 12 weeks. Serum ALT levels were decreased by 6.86, 0.54, 9.68 and 8.18% for the ML, MM, MH and STAT groups, respectively. However, these decreases were not significant. Serum alkaline phosphatase (ALP) levels showed no significant change in all rat groups.

### Effect of high fat diet consumption and Maoberry extract or STAT on fat droplets accumulation in liver tissues

**Fig. 4:**
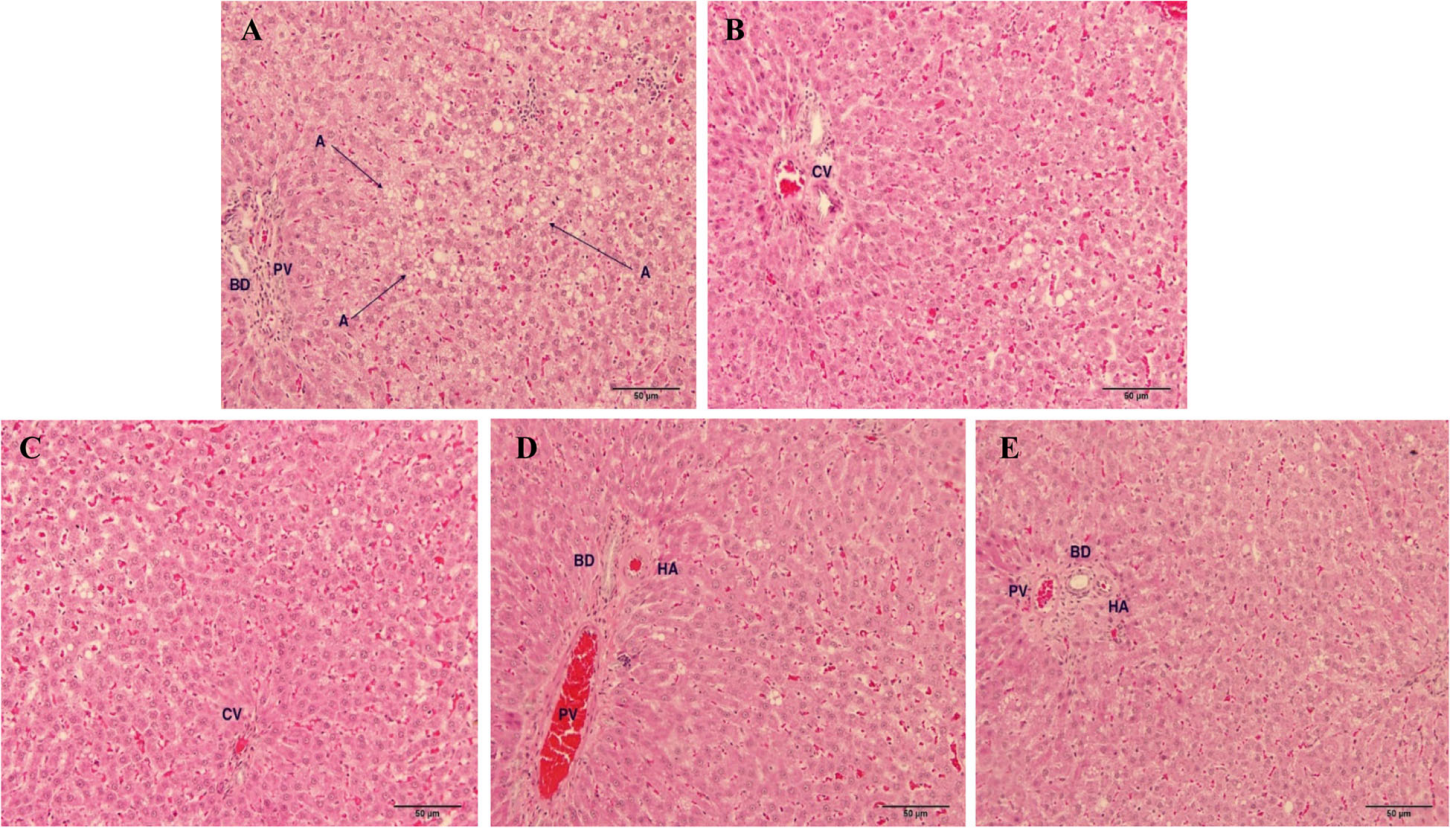
Light microscopic pathology of liver tissue of rat. H&E stain with magnification of × 20 of objective lens. **a** Rat in HF group. AA, fat droplets accumulation, **b** Rat in ML group fed for 12 weeks. **c** Rat in MM group. **d** Rat in MH group. **e** Rat in STAT group. Central vein (CV), portal vein (PV), hepatic artery (HA), bile duct (BD)

**Fig. 5:**
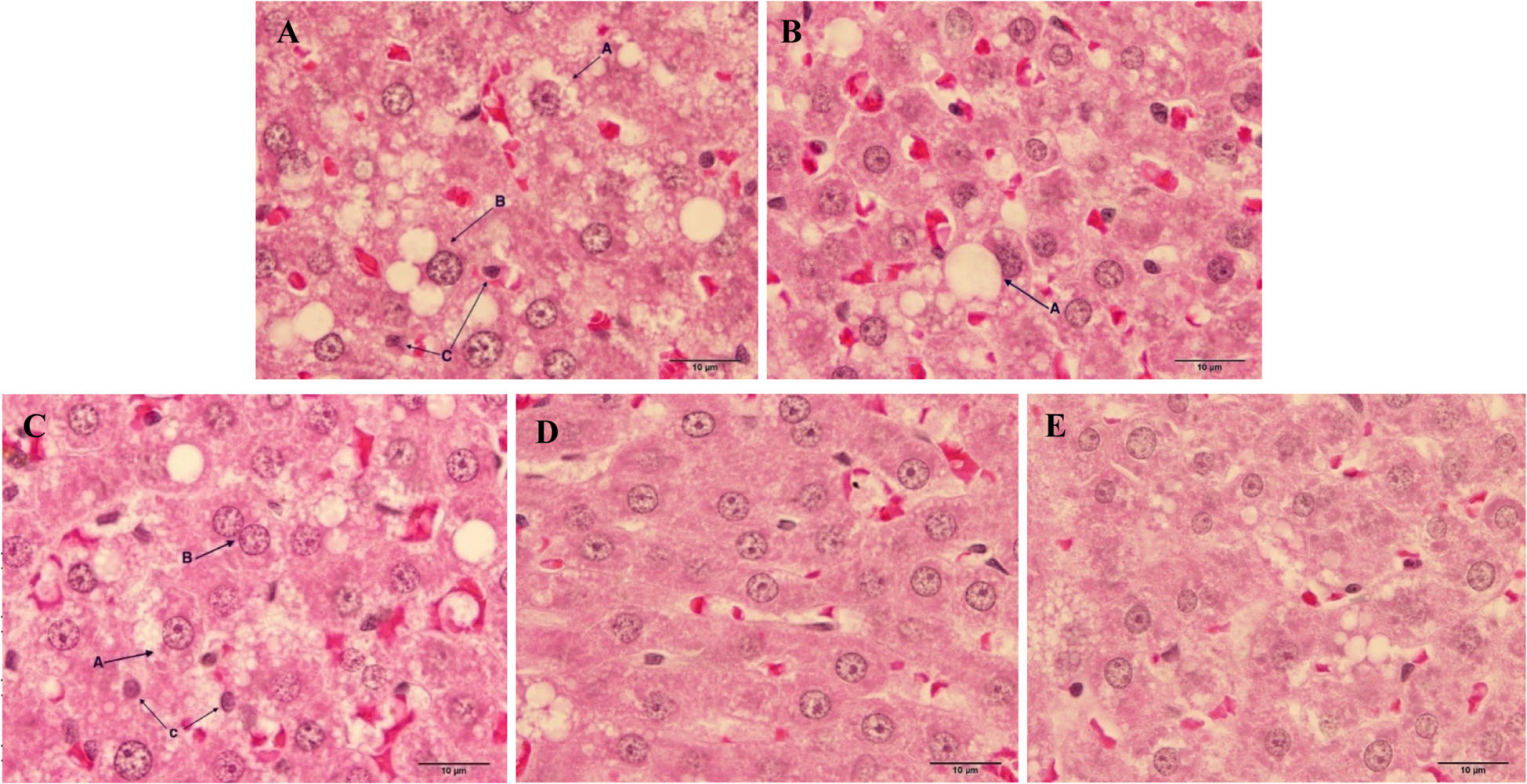
Light microscopic pathology of liver tissue of rat. H&E stain with magnification of × 100 of objective lens. **a** Rat in HF group. AA, microvesicular steatosis; AB, macrovesicular steatosis; AC, pyknotic nucleus (nucleus condensation). **b** Rat in ML group for 12 weeks. BA, macrovesicular steatosis. **c** Rat in MM group. CA, microvesicular steatosis; CB, binuclear hepatocyte and karyorrhexis nucleus (nuclear fragmentation); CC, pyknotic nucleus (nucleus condensation). **d** Rat fed in MH group. **e** Rat in STAT group

Gross observation of the whole livers in each group showed normal size, shape and colour. The surface of the liver was smooth and shiny without scar. Interestingly, foamy appearances, hepatocyte nuclei condensation and change in morphology of nucleus membrane and/or nuclear fragmentation were observed in the hepatocytes of the HF group after continuous feeding of a high fat diet for 16 weeks (Figs. 4, 5). Moreover, the pathological observation in the hepatocytes of the HF group also showed macro- and microvesicular steatosis. Histopathological features of NAFLD were found in the hepatocytes not only of the HF group but also of the ML and MM groups. The livers of rats fed high dosages of Maoberry juice and STAT for 12 weeks obviously showed diminishing numbers and size of fat droplets compared with livers of the HF group.

## Discussion

This current study focus on the liver tissue of rats which are induced to accumulate triglyceride by feeding high fat diet. Nonalcoholic fatty liver disease (NAFLD) is the accumulation of fat or triglyceride in the liver, in the absence of heavy alcohol use [6]. Pathogenic genesis to develop the accumulation of fat in the liver has found to link with many risk factors such as obesity, metabolic syndrome, excess calories, lipotoxicity, and oxidative stress [6]. There is a need to imitate the pathogenic and histological features of triglyceride accumulation be similar to human. It is very complex mechanisms in humans. This is very difficult to obtained in vitro study. Thus, rodents have mostly been used as experimental models of NAFLD to resemble the pathogenic and histological features of NAFLD [23, 24].

The environmental conditions are controlled in order to reduce or get rid of animal distress. Our previous studies reported that Maoberry extract could benefit against hypercholesterolemia and cardiovascular disease risk factors by antioxidant and anti-inflammation properties [8, 9]. The current study further demonstrates the contributions of Maoberry extract on fat metabolism in liver tissue. NAFLD has been known to be associated with obesity and metabolic syndrome. Consumption of a high fat diet causes increasing body weight and enlargement of internal organs, including the liver, as well as dyslipidemia and liver steatosis [6, 7]. After 16 weeks of high fat diet consumption, pathological analysis also showed liver steatosis and numerous fat droplets. A high fat diet commonly has been used for induction of NAFLD in rodents. This model very closely resembles the histological pattern of liver steatosis in humans [25]. Consumption of a high fat diet causes fat accumulation and cell necrosis in the liver [26, 27], as demonstrated in our histopathological study. Pyknotic and karyorrhexis nuclei are examples of cell necrosis, which is represented as a form of cell injury and death [26]. Moreover, we found macro- and microvesicular steatosis in the hepatocytes of rats fed a high fat diet. The most common histologic feature of NAFLD is steatosis, typically macro- and microvesicular steatosis [27]. Macrovesicular steatosis, which is the most common form, is characterised by large, clear fat globules accumulated in the cytoplasm of hepatocytes. The nucleus of hepatocytes is squeezed and dislocated into the rim of the cytoplasm by the fat vacuole. On the other hand, microvesicular steatosis, the hallmark of more severe hepatic dysfunction, is characterised by numerous tiny collections of foamy cells and fat droplets within the hepatocytes. Macro- and microvesicular steatosis, together with another histologic feature, such as ballooning degeneration and inflammation, may contribute to more severe disease, for instance nonalcoholic steatohepatitis and fibrosis, respectively [27, 28]. The livers of rats fed STAT as well as all three dosages of Maoberry extract, (especially the MH group) showed improvement in fat accumulation. Similar to our study, in a clinical study of 20 patients with metabolic syndrome, Kargiotis et al. [29] found that STAT monotherapy (10 mg/day) showed reduced liver steatosis on liver ultrasonography and biopsy within 12 months of treatment. In addition, previous studies have shown that supplementation of fruit extracts that mainly contained polyphenol substances (including rutin, myricetin, morin and quercetin), such as acai berry and mulberry fruit, improved the pathology of liver steatosis in rats and mice fed high fat diets [11, 30, 31]. Moreover, liver TG levels in the rats were significantly decreased with Maoberry extract in MH groups as well. Yang et al. [19] reported that consumption of a freeze-dried powder of mulberry fruit consumption showed a significant decrease in liver TG in rats with hyperlipidaemia. These beneficial effects might be due to the antioxidant power of phytochemical constituents in mulberry fruits. However, freeze-dried Maoberry was not employed in our study. This could be one of possible limitations that may affect our study. Freezed dried Maoberry might ameliorate adverse effects of lipid metabolism more more apparent. Freeze drying is used for food preservation*. Previous study has shown that* freez dried blueberries stored for 3 months were able to maintain the levels of anthocyanins and antioxidant activity as fresh blueberries [32].

In our study, rats fed a high fat diet without treatment (HF group) showed a significant increase in serum ALT levels, which may indicate impaired liver function. AST and ALT are liver enzymes that have been referred for liver disease diagnosis and assessment [33, 34]. ALT is found mostly in the cytosol of hepatocytes, but it is found less in kidneys, heart and skeletal muscle cells. However, AST is found in several cells, including hepatocytes, cardiac muscle cells, skeletal muscle cells, kidneys, brain and red blood cells [34]. When there is hepatocellular injury or death, ALT from damaged liver cells will be released into the circulation [33]. Measurement of serum ALT and/or AST concentrations, as well as body mass index, liver computed tomography and ultrasound have been used to predict the progression of NAFLD [34]. Our study showed that 12 weeks of oral gavage administration with the various dosages of Maoberry extract, resulted in lower serum ALT levels. This finding is similar to that of Guerra et al. [11]. They administered acai aqueous extract to mice fed a high fat diet for 12 weeks and found a significant decrease in liver TG contents and serum ALT levels but not serum AST levels compared with untreated mice. Moreover, acai extract improved the morphology and pathology of the liver. They suggested that the liver improvement was due to the antioxidant activity of acai extract. This may be linked to and explain our results using Maoberry, which has a high antioxidant capacity.

The purple-black fruits are often rich in anthocyanins and phenolic compounds, which are known as potent antioxidants. Our results showed the significant amount of cyanidin and peonidin in Maoberry extract. This is consistent with the study of berry fruits that reported the highest amount of cyanidin and peonidin among all anthocyanins [35]. Previously, we have reported that Maoberry extracts are sources of many essential nutrients and high in antioxidants [9]. Other bioactive compounds in Maoberry fruit (*Antidesma bunius* (L.) Spreng have been reported such as ascorbic acid, gallic acid, (−)-epicatechin, (+)-catechin, and cyanidin-3-O-glucoside [13, 36]. *Synergistic* interactions of those active ingredients available in Maobery extract have the opportunity to improve the fat metabolism in the liver tissues fed a high-fat diet. In addition to improving fat metabolism, Maoberry extract from our previous studies also showed a reduction of oxidative stress and inflammation in heart tissues [8]. Therfore, this can confirm the ability of strong antioxidant activity that is possessed by Maoberry extract.

Our other results also support the antioxidant potential of Maoberry extract by showing that 12 weeks of continuous feeding of Maoberry extract in MH groups showed significantly decreased liver TBARS levels in rats fed a high fat diet. MDA, which is a naturally-occurring oxidative product of lipid peroxidation of polyunsaturated fatty acid, was determined in the TBARS assay. MDA has been used widely as a convenient and reliable biomarker to indicate oxidative stress in cells and tissue [37, 38]. A high fat diet, especially one rich in saturated fatty acid, can induce oxidative stress for many different reasons, including increasing reactive oxygen species (ROS) production, apoptosis induction by release of cytochrome C and activation of caspases-3, which are involved in the cleavage of many key cellular proteins and DNA fragmentation, and liver injury [39]. Many studies have reported that the consumption of dietary antioxidants, particularly dietary flavonols, can improve oxidative stress markers. Lin et al. [40]. found that a high fat diet in hamsters increased liver TBARS levels, which were significantly decreased by feeding noni juice for 6 weeks. Yang et al. [19] also reported that giving mulberry fruit with a high fat diet resulted in significantly decreased liver TBARS levels due to the potential antiperoxidative agent as an antioxidant in mulberry fruit. These findings may indicate that Maoberry extract, which contains high amounts of polyphenol and flavonoid compounds, had a potential effect against oxidative stress.

We observed that Maoberry supplementation reduced expression of oxidative stress and inflammatory markers, as demonstrated by our results of TBARS assay confirming the antioxidant and anti-inflammatory properties. Many studies have reported that fruit extract treatments have a positive effect on the TNF-α status in humans and rodents with NAFLD [12, 40]. Furthermore, a previous study reported that consumption of strawberry, polyphenolic- and antioxidant-rich, in the form of a beverage for 6 weeks significantly decreased TNF-α in overweight participants in the United States [41]. Pantsulaia et al. [42] also reported the anti-inflammatory effect of citrus peel extract in mice with acute liver injury by decreasing TNF-α and interferon-γ (IFN-γ) levels. Similarly, our study indicated that Maoberry juice and STAT treatments downregulated mRNA expression of TNF-α compared with the HF group without treatment.

In addition, we measured gene expressions of GPAT-1, ACC and SREBP-1c to better understand the mechanism of the lipid-lowering pathway. Excessive free fatty acid from high fat diet consumption can cause fat droplets to accumulate in the liver because increasing free fatty acid and TG synthesis occurred [43]. ACC and GPAT-1 are key enzymes of lipid production [44]. Our study indicated that administration of Maoberry juice in rats fed a high fat diet downregulated the gene expression of ACC and GPAT-1. These findings are confirmed by the decreased liver TG levels. A previous study also reported that *Rosa laevigata* Michx, which contains a high amount of dietary flavonoids in the fruit, suppressed mRNA expression of GPAT-1 and ACC after 8 weeks of treatment in rats fed a high fat diet [30]. Therefore, our finding may be due to the antioxidant properties of polyphenol compounds and flavonoids, which occur in high levels in the fruit.

However, different foods available to human, variance of gene, species and gender as well as pathophysiology of disease in rats are quite different from human [45]. This could be limited to translate the results of animal model in this study to understand and treat in NAFLD patients.

## Conclusion

After 12 weeks of experimental study, compared to liver tissues in HF groups, the significantly different improvements of fat metabolism in MH groups were observed in the markers of liver triglyceride levels, fat accumulation in liver pathology, and gene expression of key enzymes of lipid production (GPAR-1 & ACC) anti-inflammation (TNF-alpha & IL-6). The underline mechanism that might link to fat metabolism might be through the process of antioxidant activity and bioactive ingredients in Maoberry extract accompanied with down-regulated the gene expression of key enzymes of lipid production and anti-inflammation. The exact molecular mechanism on fat metabolisms are still uncertain and complicated. Further carefully designed *studies* are need to access and clarify in human.

## Data Availability

The datasets used and/or analysed during the current study are available from the corresponding author on reasonable request.

## Abbreviations

ACC: Acetyl-CoA carboxylase
AlCl_3_: Aluminium chloride
ALT: Alanine transaminase
Cp: Crossing point
FTM-ACUC: Faculty of Tropical Medicine – Animal Care and Use Committee
g: Gram
GAE: Gallic acid equivalents
GPAT-1: Glycerol-3-phosphate acyltransferase 1
HCl: *Hydrochloric* acid
HF: A high-fat
HPLC: High performance liquid chromatography
IL-6: Interleukin-6
kcal: Kilocalorie
M H: High dosage of Maoberry extract
M: Molar
MDA: Malondialdehyde
ML: Low dosage of Maoberry extract
mL: Melilite
MM: Medium dosage of Maoberry extract
mRNA: Messenger RNA;
NAFLD: Non-alcoholic fatty liver disease
NaNO_2_: Sodium nitrite
NaOH: Sodium hydroxide
RT-qPCR: *Real*-time polymerase chain reaction
SD: Sprague-Dawley
SD: Standard deviation
SREBP-1c: Sterol regulatory element-binding transcription factor 1
STAT: Statin
TBARS: Thiobarbituric acid reactive substance
Tm: Melting temperature
TNF-α: *Tumor necrosis factor* ailp
μL: Microlite
μm: Micrometre
